# The gene expression profiles of canine mammary cancer cells grown with carcinoma-associated fibroblasts (CAFs) as a co-culture *in vitro*

**DOI:** 10.1186/1746-6148-8-35

**Published:** 2012-03-27

**Authors:** Magdalena Król, Karol M Pawłowski, Katarzyna Szyszko, Henryk Maciejewski, Izabella Dolka, Elisabetta Manuali, Michał Jank, Tomasz Motyl

**Affiliations:** 1 Department of Physiological Sciences, Faculty of Veterinary Medicine, Warsaw University of Life Sciences - WULS, Nowoursynowska 159, 02-776 Warsaw, Poland; 2 Department of Animal Environment Biology, Faculty of Animal Sciences, Warsaw University of Life Sciences - WULS, Ciszewskiego 8, 02-786 Warsaw, Poland; 3 Institute of Computer Engineering, Control and Robotics I-6, Wroclaw University of Technology, Wybrzeże Wyspiańskiego 27, 50-320 Wroclaw, Poland; 4 Department of Pathology and Veterinary Diagnostics, Faculty of Veterinary Medicine, Warsaw University of Life Sciences - WULS, Nowoursynowska 159, 02-776 Warsaw, Poland; 5 Area Diagnostica Integrata Istologia e Microscopia Elettronica Istituto Zooprofilattico Sperimentale dell'Umbria e delle Marche, Via G. Salvemini 1, 06126 Perugia, Italy

## Abstract

**Background:**

It is supposed that fibroblasts present in tumour microenvironment increase cancer invasiveness and its ability to metastasize but the mechanisms have not been clearly defined yet. Thus, the current study was designed to assess changes in gene expression in five various cancer cell lines grown as a co-culture with the carcinoma-associated fibroblasts (CAFs) *in vitro*.

**Results:**

A carcinoma-associated fibroblast cell line was isolated from a canine mammary cancer. Then, a co-culture of cancer cells with the CAFs was established and maintained for 72 hrs. Having sorted the cells, a global gene expression in cancer cells using DNA microarrays was examined. The analysis revealed an up-regulation of 100 genes and a down-regulation of 106 genes in the cancer cells grown as a co-culture with the CAFs in comparison to control conditions. The PANTHER binomial statistics tool was applied to determine statistically over-manifested pathways (*p *< 0.05). Bulk of the up-regulated genes are involved in the adhesion, the angiogenesis, the epithelial-mesenchymal transition (EMT) and generally take part in the developmental processes. These results were further confirmed using real-time qPCR. Moreover, a wound-healing assay and growth characteristics on Matrigel matrix showed that CAFs increase cancer cell migration and matrix invasion.

**Conclusion:**

The results of the current study showed that the co-culturing of cancer cells and the CAFs caused significant changes to the cancer gene expression. The presence of the CAFs in a microenvironment of cancer cells promotes adhesion, angiogenesis and EMT.

## Background

Since canine mammary tumours in bulk are of epithelial origin this kind of cells is subjected to many studies. Over the last few years it has also been pin-pointed that concomitant changes occur within stromal cells, which contribute to the tumour microenvironment as well [1,2]. Tumour microenvironment embraces inflammatory, fibroblastic, endothelial cells, adipocytes and other. Changes within these stromal cells have been postulated to increase the tumorigenic phenotype of the epithelial cell, promote malignant transformation, induce epithelial-mesenchymal transition (EMT) and promote tumour spreading and metastasis [3]. It is worth noting however, that in almost all the tumours, the main cell type of cancer stromal compartment is fibroblast. These cells are usually atypical and are termed carcinoma-associated fibroblasts (CAFs). We assume there is a cross-talk between the tumour cells and the CAFs, which promotes migratory, and invasive properties of cancer cells [3] though their exact role within cancer microenvironment has not been fully defined yet. Thus, the study was conducted to assess the changes in gene expression in cancer cells grown as a co-culture with the CAFs *in vitro*. As far as we know the study presented hereby is a pioneering microarray experiment in this field. Despite that our study involved five various cell lines, only one CAFs cell line was used, thus the results may be limited to this particular CAF model. Further studies in this field are required.

The analysis revealed an up-regulation within a span of 100 genes and a down-regulation within 106 genes in cancer cells grown as a co-culture with the CAFs, comparing against set control conditions. In this manuscript we focused mainly on the gene sets involved in adhesion, developmental process and neurotransmissions.

The results of our study can be extrapolated on human research because canine mammary tumours are being considered a spontaneous animal model of human breast cancer [4]. There are many similarities between human and canine mammary cancers: in both species they represent a heterogeneous group in terms of morphology and biological behaviour [5], in both similar cancer-related pathways are activated [6-8] as much as both species live under similar environmental conditions.

## Methods

### Cell lines

The cell lines used for this study have previously been given an account of [9-12]. Two canine mammary adenocarcinoma cell lines (CMT-W1, CMT-W2), an anaplastic cancer cell line (P114), a simple carcinoma cell line (CMT-U27) and a spindle-cell mammary tumour cell line (CMT-U309) were examined. The CMT-W1 and the CMT-W2 cell lines had kindly been donated by Prof. Dr. Maciej Ugorski and Dr. Joanna Polanska from Wroclaw University of Environmental and Life Sciences (Poland). The CMT-U27 cell line had kindly been donated by Dr. Eva Hellmen from Swedish University of Agricultural Sciences (Sweden) and the P114 cell line had kindly been donated by Dr. Gerard Rutteman from Utrecht University (The Netherlands).

The cells were cultured under optimal conditions: a medium (RPMI-1640) enriched with 10% (v/v) heat-inactivated fetal bovine serum (FBS), penicillin-streptomycin (50 iU mL-1), and fungizone (2.5 mg mL-1) (reagents obtained from Sigma Aldrich, USA), in an atmosphere of 5% CO2 and 95% humidified air at 37°C, and routinely sub-cultured every other day. The methods of canine mammary cancer cells culturing have previously been given an account of [9-12].

### Tumour sample

A mammary tumour was surgically removed during mastectomy on a 12 years old mixed breeds female. The tumour then, was divided into equal halves, one of them was fixed in 10% neutral buffered formalin and routinely embedded in paraffin to perform histological assay. The other, was used to isolate and establish a carcinoma-associated fibroblast cell line.

### Carcinoma-associated fibroblasts isolation

The cells isolation from cancer tissue has been described in our previous manuscript [13]. The tumor sample was collected into the medium RPMI 1640 (Sigma Aldrich, USA) containing flask immediately after mastectomy. The RPMI 1640 medium had been used to maintain the same culturing conditions for mono- and co-culture. The tumour sample was then sliced and cultured overnight in collagenase containing medium RPMI 1640 according to the Limon et al. [14] protocol (modified by Dr Eva Hellmen, Swedish University of Agricultural Sciences, Sweden). The following day, the medium was centrifuged and pellet was suspended in a fresh culture medium supplemented in FGF (10 nM/ml, obtained from Sigma Aldrich, USA), a medium that encourages preferential fibroblastic outgrowth.

### Histopathological examination

The tissue sample embedded in paraffin block was cut into five μm sections and baked in 37°C overnight. After dewaxing in xylene and rehydration in ethanol, for antigen retrieval, the slides were placed in 0.02 M citrate buffer, pH 6.0 and boiled in the decloaking chamber. The tumor type was classified based on the World Health Organization (WHO) Histological Classification and Mammary Tumors of the Dog and Cat classification [15,16]. The mammary carcinoma grading was assessed in respect to tubule formation, degree of differentiation and mitotic index.

The carcinoma-associated fibroblasts and the canine mammary cancer cells were cultured on Lab-Tek (Nunc Inc., USA) 4-chamber culture slides and were then fixed with ethanol after the 24 hrs.

The immunohistochemical examination of expression of Ki67, cytokeratin, vimentin, smooth muscle actin, s100 protein, p63 protein was performed on the tissue sample as well as on carcinoma-associated fibroblasts to confirm the origin of cell culture. The MUC1 expression was analyzed in the canine mammary cancer cell lines.

The samples were incubated in the Peroxidase Blocking Reagent (Dako, Denmark) for 10 min at room temperature prior to the antibody incubation. After 30 min incubation in 5% bovine serum albumin (Sigma Aldrich, Germany), the following primary antibodies were used (diluted in 1% bovine serum): mouse monoclonal anti-Ki67 (Clone MIB-1) at the concentration 1:75; monoclonal mouse anti-human cytokeratin (Clone MNF116) at the concentration 1:50; monoclonal mouse anti-human vimentin (Clone Vim 3B4) at the concentration 1:50; monoclonal mouse anti-human actin (Clone HHF35) at the concentration 1:50; polyclonal rabbit anti-S100 (ready to use solution) all obtained from Dako (Denmark); monoclonal mouse anti-p63 protein (Santa Cruz Biotechnology, USA) and monoclonal mouse anti-MUC1 (Abcam, United Kindgdom) at the concentration 1:10. According to the manufacturer's instructions the slides were incubated with antibodies at +4°C overnight or 1 hr at room temperature. For the staining the anti-mouse or anti-rabbit EnVision kits (Labelled Polymers consist of secondary anti-rabbit antibodies conjugated with the HRP enzyme complex obtained from Dako) were used. To develop the coloured product, the 3,3'-Diaminobenzidine (DAB) substrate was used (Dako). Finally, the haematoxylin was used for nuclei counterstaining.

For each immunohistochemical analysis as the negative control, the staining without the use of primary antibodies was done. The pictures were taken using Olympus microscopy BX60 (Olympus, Germany).

### Co-culture and sorting

The CAFs (10^5 ^cells) were grown on 75 cm^2 ^culture flasks and the cancer cells (CMT-W1, CMT-W2, CMT-U27, CMT-U309, P114) were layered (5 × 10^5 ^cells) on the top of the CAFs (fibroblasts and cancer cells at 1:5 ratio [17]). An Orange CellTracker fluorescent dye CMTMR (Invitrogen, USA) was used to stain the CAFs' population before the cancer cell population was added. Initially, optimal staining conditions were determined by incubating CAFs in various concentrations of CMTMR (5-25 μM dye, according to the manufacturer's instructions) and checking the fluorescence signal after 72 hrs using FACS. The lowest concentration that gives positive results has been used in further experiments (5 μM). Staining was accomplished by incubation in serum/antibiotics-free RPMI medium containing 5 μM CMTMR (10 mM stock in DMSO; Sigma Aldrich, USA) for 45 min at 37°C. Subsequently, the medium was aspirated, and the CAFs were washed with PBS twice and incubated with complete RPMI for 1 hr and then again washed to remove any remnant non-metabolized CMTMR. The cancer cells were placed on the CMTMR-stained CAFs.

The co-culture was maintained for 72 hrs. Then, the cells were harvested by trypsynization, analyzed and sorted using FACS Aria II high speed cell sorter with Diva 5.0 software (Becton Dickinson, USA). Based on the FSC and SSC cytogram, live cells were gated to exclude all dead cells, cell debris and cell clumps. Within the gated cell populations, fluorescing cells were identified as CMTMR-labelled carcinoma-associated fibroblasts and non-fluorescent as cancer cells. Excitation wavelength used was 488 nm, whereas emission wavelength used was 578 nm. Cancer cells were sorted into RPMI 1640 medium in 15 ml polypropylene tubes (BD Biosciences).

### Confocal microscopy

The CAFs grown as a mono-culture were stained using Orange CellTracker fluorescent dye CMTMR, as described above. The cells grown on plastic were fixed in 70% ethanol (10 min), washed in PBS three times and the coverslips were mounted on microscope slides using ICN mounting medium. The cell imaging was performed on confocal laser scanning microscope FV-500 system (Olympus Optical Co, Germany) after 1 hr, then after 72 hrs after the staining. The excitation/emission were: HeNe 543 nm laser with 610 nm filter for CMTMR staining. The cells were examined using the Fluoview program (Olympus Optical Co., Germany). The pictures have been analyzed using a computer-assisted image analyzer (Olympus Microimage™ Image Analysis, software version 4.0 for Windows, USA).

### Wound-healing assay

To assess the migration ability of cancer cells grown as a co-culture with CAFs, we applied a wound-healing test. The cancer cells (grown as the co-culture with CAFs at the 5:1 ratio, and normal control cells) were separately seeded in multi-well plates and then, (after 72 hrs when the cells were confluent) using a pipette tip (100 ul) a straight scratch had been made, simulating a wound. The images were captured at the beginning and at regular intervals (after 2, 4 and 6 hours) during cell migration to close the wound. The images then were compared to quantify the cells' migration rate. This method is particularly suitable for studies of cell-cell interaction on cell migration [18]. The pictures have been analyzed using a computer-assisted image analyzer (Olympus Microimage™ Image Analysis, software version 4.0 for Windows, USA).

### 3D culture

Cancer cells were treated with trypsin and resuspended in culture medium. 35 mm culture plates (Corning Inc.) were coated with 100 μl of growth factor reduced Matrigel (BD Biosciences) and left to solidify for 30 min. at 37°C. The control cells were then plated at a concentration of 10^4 ^cells/ml. Co-cultured cells were plated at the same concentration (cancer cells and CAFs at 5:1 ratio). The growth of cells on Matrigel was observed everyday under phase-contrast microscope (Olympus).

### Microarray analysis

The sorted cancer cells grown as a co-culture were centrifuged (2,500 rpm for 5 min), whereas cancer cells grown as mono-cultures were washed with PBS and harvested by trypsynization and centrifuged (2,500 rpm for 5 min). The total RNA from the samples was isolated using a Total RNA kit (A&A Biotechnology, Poland) according to the manufacturer's protocol. The isolated RNA samples were dissolved in RNase-free water. The quantity of the isolated RNA was measured using NanoDrop (NanoDrop Technologies, USA). The samples with adequate amounts of RNA were treated with DNaseI to eliminate DNA contamination. The samples were subsequently purified using RNeasy MiniElute Cleanup Kit (Qiagen, Germany). Finally the RNA samples were analyzed on a BioAnalyzer (Agilent, USA) to measure the final RNA quality and integrity.

The total RNA (10 μg) of each cell line was reverse-transcribed using SuperScript Plus Direct cDNA Labeling System, (Invitrogen, USA) according to the manufacturer's protocol for each microarray slide. Single-strand cDNAs were stained with Alexa 647 and Alexa 555 (Invitrogen). Dog-specific oligonucleotide microarray slides *Canis familiaris *V1.0.1 AROS (Operon, USA) with 25,383 probes were used for the hybrydization. Hybridization was performed using automatic hybridization station HybArray12 (PerkinElmer, USA). Two replicates were made (dye-swap).

The slides were analyzed using microarray scanner ScanArray HT and ScanExpress software (PerkinElmer, USA).

### Real-time qPCR

The mRNA sequences of the key genes were obtained from NCBI database. Primers were designed using PRIMER3 software (free on-line access) and checked using Oligo Calculator (free on-line access) and Primer-Blast (NCBI database). Primers' sequences are listed in Table 1. *HPRT *and *RPS19 *genes were used as non-regulated reference genes for normalization of target gene expression [19,20]. Quantitative RT-PCR was performed using fluorogenic Lightcycler Fast Strand DNA Sybr Green (Roche) and the Light Cycler (Roche). The results were analyzed using comparative Ct method [21]. Relative transcript abundance of the gene equals ΔCt values (ΔCt = Ct^reference ^- Ct^target^). Relative changes in transcript are expressed as ΔΔCt values (ΔΔCt = ΔCt^co-culture ^- ΔCt^control^). The experiment was conducted three times.

**Table 1:** Primers used for Real-time qPCR

Gene symbol	Forward primer	Reverse primer	Optimum annealing temp. (°C)	Optimum annealing time (sec)
*DSP*	CAGACTCACCGAAGAGGAAA	CTGCTGTGAAGTCTGGGAGT	61	7

*MAG*	TGCCATCGTCTGCTACATTA	CAGTCGCCTCTCACTCTCAT	60	6

*PCDH19*	CTTTCACATCACTGCACTCG	GTGTGTTGGGAGGTGAGTTC	61	6

*HPRT*	AGCTTGCTGGTGAAAAGGAC	TTATAGTCAAGGGCATATCC	59	6

*RPS19*	CCTTCCTCAAAAAGTCTGGG	GTTCTCATCGTAGGGAGCAAG	61	10

Primers sequences used in this study and their annealing optimal temperature and time. The mRNA sequences of key genes were obtained from NCBI database. Primers were designed using PRIMER3 software (free on-line access) and checked using Oligo Calculator (free on-line access) and Primer-Blast (NCBI database). *HPRT *and *RPS19 *genes were used as non-regulated reference genes for normalization of target gene expression [19,20].

### Statistical analysis

In the analysis of differential gene expression, background-corrected value of signal in each microarray channel was used. Prior to the analysis, non-specific filtering was performed, i.e. genes with small level of expression were removed (we set an arbitrary threshold according to which at least half of the samples' expression was to be at least 100). This reduced the number of genes down to 24 842. Then the log2 ratio of the sample vs control channels was calculated and the signal was loes normalized. Quality control, including MA analysis, and signal normalization were done with the Bioconductor software. The analysis of differential expression was performed using linear methods for microarrays (limma package in Bioconductor software) [22]. The method tests the null hypothesis of no differential expression between the sample and control groups using the moderated t-statistic [22], which has similar interpretation as the ordinary *t*-test statistic. We identified 206 genes with the p-value below 0.05 and fold change > 2.0.

The microarray data discussed in this publication has been deposited in NCBI's Gene Expression Omnibus and is freely accessible through GEO Series accession number GSE29601.

The gene function was identified using the NCBI database and PANTHER pathway analysis software [23]. The pathway analyses were conducted using binominal statistic test (PANTHER) with the cut-off value *p *< 0.05.

The statistical analysis of optical density, wound healing assay and Real-time qPCR was conducted using Prism version 5.00 software (GraphPad Software, USA). The one-way ANOVA, and ANOVA + Tukey HSD (Honestly Significant Difference) post-hoc test as well as *t*-test were applied. The p-value < 0.05 was regarded as significant whereas p-value < 0.01 and p-value < 0.001 as highly significant.

## Results

### Tumor sample and CAFs examination

**Figure 1:**
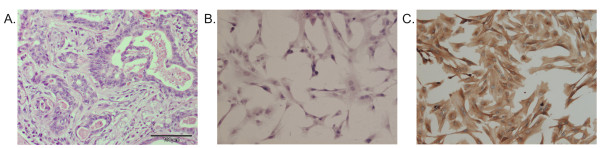
**Cancer associated fibroblasts isolated from canine mammary cancer**. Representative pictures of A. Canine mammary complex carcinoma (HE staining) tissue from which the carcinoma-associated fibroblasts were isolated. B. The culture of carcinoma-associated fibroblasts (CAFs) isolated from the canine mammary complex carcinoma (HE staining) and C. Carcinoma-associated fibroblasts (CAFs) isolated from canine complex mammary carcinoma revealed a strong vimentin expression (brown color). The pictures were obtained using Olympus BX60 microscope (at the magnification of 200×).

The histopathological and immunohistochemical assessment of the tissue sample, from which the carcinoma-associated fibroblasts were isolated, showed that the tumour type was a complex carcinoma of the 1^st ^grade malignancy (Figure 1a). The histopathological and immunohistochemical analysis of the isolated cells (Figure 1b) revealed that the cell line did not express cytokeratin, S100 protein, p63 protein and actin, whereas a strong vimentin expression was detected (at the level of 3 in 0-3 scale) (Figure 1c). The analysis confirmed that the isolated cell line is an atypical colony of fibroblasts which are termed the CAFs.

### Sorting of the co-cultured cells

**Figure 2:**
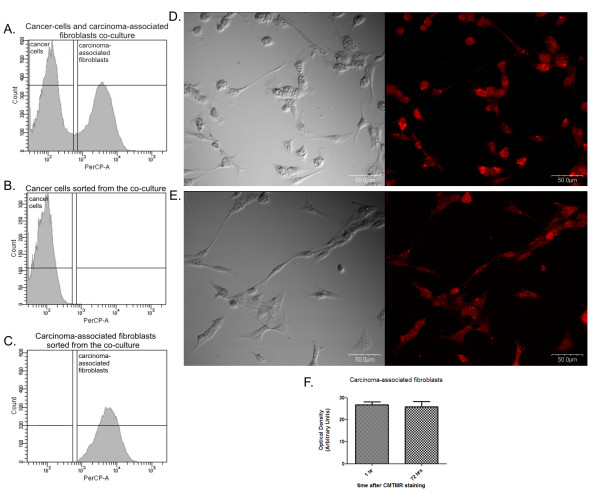
**Histogram and pictures of unstained cancer cells and stained CAFs**. A. Representative histogram and sorting gates of unstained cancer cells and CMTMR-stained carcinoma-associated fibroblasts (CAFs) grown as the co-culture for 72 hrs. B. Histogram of cancer cells sorted from co-culture showing only CMTMR-negative cells. C. Histogram of CAFs sorted from co-culture showing only CMTMR-positive cells. D. and E. Representative pictures obtained using confocal microscopy showing red-colored stained cytoplasm (CMTMR staining) of CAFs growing as a single culture for 1 hr and 72 hrs, respectively. The imaging of cells was performed on confocal laser scanning microscope FV-500 system (Olympus Optical Co, Germany). The cells were examined using the Fluoview program (Olympus Optical Co., Germany). F. The graph showing mean optical density of the red (reflecting CMTMR staining) in CAFs at 1 hr and 72 hrs after staining. For statistical purposes the *t*-test has been applied (Graph Pad 5.0), no significant difference has been observed between these two values.

The Flow-Cytometry easily distinguished the CMTMR-stained cells from the unstained ones (Figure 2a) and allowed the proper further sorting of each population (Figure 2b, c). The co-culture was maintained for 72 hrs. According to the manufacturer's instruction, CMTMR probes remain vividly fluorescent for at least 72 hrs after incubation in fresh medium at 37°C and through at least four cell divisions. The confocal observations confirmed that after 72 hrs of the staining with the CMTMR all the cells showed red-staining cytoplasm pattern (Figure 2c, d, e). No detrimental effects on proliferation and plating efficiency was observed. Our analysis of optical density of the red dye in the cells measured 1 hr and 72 hrs after the staining showed very similar results (any statistically significant difference had not been found) (Figure 2f). It indicates that there wasn't any staining loss, thus we suppose that the dye wasn't leaking from CAFs to stain cancer cells. It had been also suggested in the subject literature [24]. Moreover, confocal microscopy analysis of the red dye fluorescence with Nomarski Interferenced Contrast showed that all of the stained CAFs maintained their staining pattern after the 72 hrs. Thus, the artificial sorting of unstained CAFs as cancer cells is not very probable. Our FACS sorting isolated a 97-99% pure population on postsort (assessed by BC FACS Diva 5.0 software) what was checked by FACS (Figure 2b, c) and fluorescence microscopy (data not shown). Previously published study of the human fibroblasts and epithelial cells sorting based on the cell tracker staining showed similar results [25].

### Migration assay and growth characteristics on Matrigel matrix

**Figure 3:**
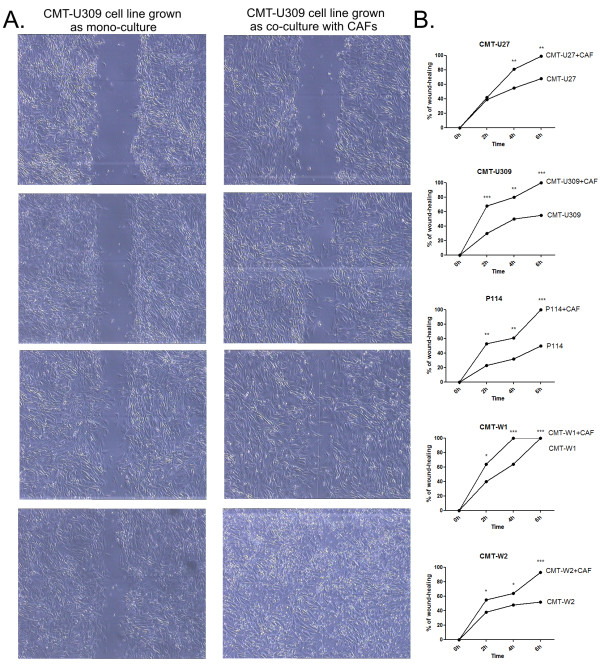
**Wound healing assay of canine mammary cancer cells grown in control conditions and as a co-culture with CAFs**. A Representative pictures of migration (wound closing) of CMT-U309 cell line grown as a mono-culture and co-culture with CAFs at 0, 2, 4 and 6 hrs after the scratch was made. B The graphs of % of wound closure after the 2, 4 and 6 hrs of migration. The pictures were taken using phase-contrast microscopy (Olympus) at the magnification of 100×. The statistical analysis was performed using Prism version 5.00 software (GraphPad Software, USA). The one-way ANOVA was applied to analyze the results. *p *< 0.05 was regarded as significant and marked as *, whereas *p *< 0.01 and *p *< 0.001 were regarded as highly significant and marked as ** and ***, respectively.

The wound healing assay showed that in all the cancer cell lines the co-culturing with CAFs increased their migratory abilities (Figure 3). CMT-U27 cells grown with CAFs almost completely closed the wound (99%) in 6 hours, whereas CMT-U27 control cells after 6 hrs closed 68% of the wound. Similarly, CMT-U309 and P114 cells (grown with CAFs) after 6 hrs completely closed the wound (100%), whereas control cells closed only 55% and 50% (respectively) of the wound. CMT-W1 cells grown with CAFs completely closed the wound after 4 hrs, whereas CMT-W1 control cells after 6 hrs (after 4 hrs 64% of the wound was closed). CMT-W2 cells grown with CAFs closed 93% of the wound after 6 hrs, whereas control cells closed only 52% of the wound.

**Figure 4:**
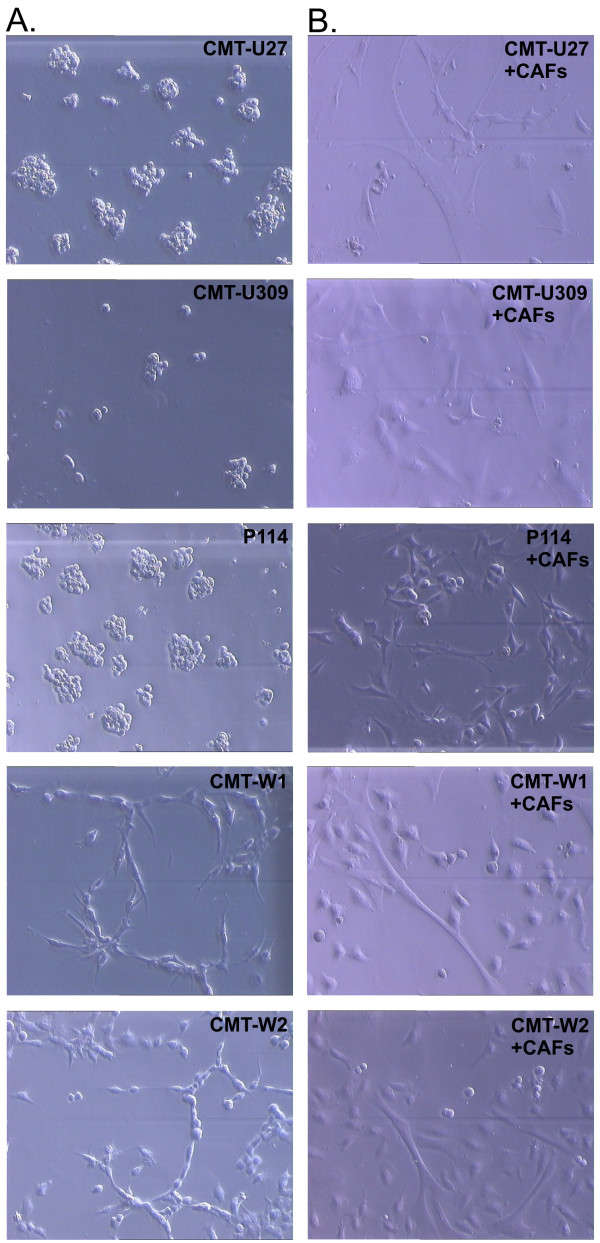
**Canine mammary cancer cell lines growth characteristics in Matrigel matrix**. A Growth characteristics of CMT-U27, CMT-U309, P114, CMT-W1 and CMT-W2 cell lines (phase contrast micrographs) grown on Matrigel matrix for 72 hours. B Growth characteristics of CMT-U27, CMT-U309, P114, CMT-W1 and CMT-W2 cell lines (phase contrast micrographs) grown as a co-culture with CAFs on Matrigel matrix for 72 hours. The pictures were taken using phase-contrast microscopy (Olympus) at the magnification of 200×.

To assess the ability of the cell lines to matrix invasion, we have assessed their growth characteristics on Matrigel matrix (Figure 4). After 72 hrs of culturing (similarly as in all experiments) on Matrigel CMT-U27, CMT-U309 and P114 cell lines formed colonies, whereas CMT-W1 and CMT-W2 cell lines formed branching structures (Figure 4a) what indicated their invasive phenotype. However, all the cell lines grown as a co-culture with CAFs after 72 hrs were dispersed (Figure 4b) what indicated increase of their matrix invasion ability by CAFs.

### Gene expression analysis

The gene expression analysis showed similar rate of gene expression in each of the dye-swap experiment. This result indicates that all microarray samples were successfully labeled, hybridized, and scanned. The discriminating analysis (with p value cut-off < 0.05; fold change > 2.0) revealed 106 up-regulated (Table 2) and 100 down-regulated (Table 3) genes in cancer cells grown as a co-culture with the CAFs in each of the slides. These up/down-regulated genes were common for each cell line examined individually, compared to the same cell line grown as a mono-culture. Thus, these genes were activated/inactivated in all of the cell lines under co-culture conditions with the CAFs.

**Table 2:** Up-regulated genes in cancer cells grown as a co-culture with CAFs

No	Fold change	Gene ID	Gene name	Molecular function	Biological process
1	4.18	TRIM6	Tripartite motif-containing protein 6	ubiquitin-protein ligase activity; structural constituent of cytoskeleton; RNA binding; cytoskeletal protein binding	spermatogenesis; neurotransmitter secretion; intracellular protein transport; exocytosis; cell cycle; signal transduction; synaptic transmission; carbohydrate metabolic process; protein metabolic process; cell-cell signaling; dorsal/ventral axis specification; mesoderm development; mammary gland development

2	3.76	PPP1R12A	Protein phosphatase 1 regulatory subunit 12A	protein binding; phosphatase regulator activity	protein metabolic process

3	3.73	TCHHL1	Trichohyalin-like protein 1		

4	3.24	**QRICH2**	Glutamine-rich protein 2	receptor activity	fertilization; **cell adhesion**

5	3.14	TMEM82	Transmembrane protein 82		

6	3.14	ZNF212	Zinc finger protein 212	DNA binding; transcription factor activity	nucleobase, nucleoside, nucleotide and nucleic acid metabolic process

7	3.13	PYROXD1	Pyridine nucleotide-disulfide oxidoreductase domain-containing protein 1	oxidoreductase activity	immune system process; respiratory electron transport chain; apoptosis; ferredoxin metabolic process; oxygen and reactive oxygen species metabolic process

8	3.10	PDE5A	cGMP-specific 3',5'-cyclic phosphodiesterase	hydrolase activity, acting on ester bonds	visual perception; sensory perception; signal transduction; nucleobase, nucleoside, nucleotide and nucleic acid metabolic process; signal transduction

9	3.02	**CHAD**	Chondroadherin	receptor activity	immune system process; cell surface receptor linked signal transduction; **cell-cell adhesion**; mesoderm development; skeletal system development

10	3.00	QSER1	Glutamine and serine-rich protein 1		

11	2.96	PRKX	Serine/threonine-protein kinase	kinase activity	muscle contraction; neurological system process; mitosis; intracellular signaling cascade; protein metabolic process; signal transduction;

12	2.90	ZFAND5	AN1-type zinc finger protein 5	nucleic acid binding	sensory perception; respiratory electron transport chain

13	2.86	C2CD3	C2 domain-containing protein 3		

14	2.82	CEP110	Centriolin;Centrosomal protein of 110 kDa		

15	2.80	**FOXQ1**	Forkhead box protein Q1	DNA binding; transcription factor activity	visual perception; sensory perception; cell cycle; cell surface receptor linked signal transduction; carbohydrate metabolic process; nucleobase, nucleoside, nucleotide and nucleic acid metabolic process; **cellular component morphogenesis**; segment specification; anterior/posterior axis specification; ectoderm development; mesoderm development; embryonic development; nervous system development

16	2.80	NUP210L	Nuclear pore membrane glycoprotein 210-like		intracellular protein transport; nuclear transport

17	2.79	SLC22A11	Solute carrier family 22 member 11	ATPase activity, coupled to transmembrane movement of substances; ligase activity; carbohydrate transmembrane transporter activity; cation transmembrane transporter activity	cation transport; anion transport; extracellular transport; carbohydrate transport; carbohydrate metabolic process

18	2.78	HERC2	Probable E3 ubiquitin-protein ligase HERC2	ubiquitin-protein ligase activity	protein metabolic process; ectoderm development; mesoderm development; skeletal system development; nervous system development

19	2.76	**PCSK6**	Proprotein convertase subtilisin/kexin type 6	peptidase activity	cell surface receptor linked signal transduction; **cell-matrix adhesion**; protein metabolic process; signal transduction; mesoderm development

20	2.76	UCK2	Uridine-cytidine kinase 2	kinase activity; transferase activity, transferring glycosyl groups	carbohydrate metabolic process; nucleobase, nucleoside, nucleotide and nucleic acid metabolic process

21	2.76	WFDC5	WAP four-disulfide core domain protein 5	protein binding;peptidase inhibitor activity	protein metabolic process

22	2.75	RPS10	40S ribosomal protein S10	structural constituent of ribosome; nucleic acid binding	protein metabolic process

23	2.75	SLC7A14	Probable cationic amino acid transporter	amino acid transmembrane transporter activity; transmembrane transporter activity	amino acid transport; cellular amino acid and derivative metabolic process

24	2.75	UBQLN2	Ubiquilin-2;UBQLN2		protein metabolic process

25	2.70	**CLEC7A**	C-type lectin domain family 7 member A	receptor activity; receptor binding	B cell mediated immunity; natural killer cell activation; intracellular protein transport; endocytosis; signal transduction; **cell-cell adhesion**; signal transduction; cellular defense response

26	2.70	**SH3BP1**	SH3 domain-binding protein 1	protein binding; small GTPase regulator activity	cell surface receptor linked signal transduction; signal transduction; **cellular component morphogenesis**

27	2.68	HMGB2	High mobility group protein B2	DNA binding; chromatin binding; receptor binding; transcription factor activity	intracellular signaling cascade; nucleobase, nucleoside, nucleotide and nucleic acid metabolic process; signal transduction; organelle organization; establishment or maintenance of chromatin architecture

28	2.68	TMSB10	Thymosin beta-10		

29	2.67	**NEFM**	Neurofilament medium polypeptide	structural constituent of cytoskeleton	**cellular component morphogenesis**; ectoderm development

30	2.67	ZNF274	Zinc finger protein 274	DNA binding;transcription factor activity	nucleobase, nucleoside, nucleotide and nucleic acid metabolic process

31	2.66	**PCDH19**	Protocadherin-19	G-protein coupled receptor activity; calcium ion binding	cell surface receptor linked signal transduction; **cell-cell adhesion**; cell motion; signal transduction; **cellular component morphogenesis**; ectoderm development; mesoderm development; embryonic development; nervous system development; heart development; muscle organ development

32	2.61	KCMF1	E3 ubiquitin-protein ligase KCMF1		

33	2.60	PIP4K2B	Phosphatidylinositol-5-phosphate 4-kinase type-2 beta	kinase activity	cell surface receptor linked signal transduction; lipid metabolic process; signal transduction

34	2.57	GNAT3	Guanine nucleotide-binding protein G(t) subunit alpha-3	GTPase activity; protein binding	cell surface receptor linked signal transduction; signal transduction

35	2.56	LPCAT1	1-acylglycerophosphocholine O-acyltransferase 1	acyltransferase activity; calcium ion binding; calmodulin binding; calcium-dependent phospholipid binding	metabolic process

36	2.56	XPR1	Xenotropic and polytropic retrovirus receptor 1	G-protein coupled receptor activity	cell surface receptor linked signal transduction; signal transduction; embryonic development

37	2.54	**LPHN2**	Latrophilin-2	G-protein coupled receptor activity	immune system process; neurotransmitter secretion; intracellular protein transport; exocytosis; cell surface receptor linked signal transduction; synaptic transmission; **cell adhesion**; cell-cell signaling; mesoderm development; angiogenesis; heart development

38	2.54	MPHOSPH6	M-phase phosphoprotein 6		cell cycle

39	2.52	NDUFB10	NADH dehydrogenase [ubiquinone] 1 beta subcomplex subunit 10	oxidoreductase activity	oxidative phosphorylation; respiratory electron transport chain

40	2.51	**DSP**	Desmoplakin	structural constituent of cytoskeleton; cytoskeletal protein binding	**cell adhesion**; **cellular component morphogenesis**; ectoderm development

41	2.51	EXOC3L2	Exocyst complex component 3-like protein 2		spermatogenesis; immune response; macrophage activation; intracellular protein transport; exocytosis; mesoderm development; angiogenesis; hemopoiesis; response to stimulus

42	2.50	UBXN1	UBX domain-containing protein 1		

43	2.49	MYOT	Myotilin;		

44	2.49	SHPRH	E3 ubiquitin-protein ligase	DNA helicase activity; nucleic acid binding	nucleobase, nucleoside, nucleotide and nucleic acid metabolic process; organelle organization; establishment or maintenance of chromatin architecture

45	2.49	SMYD1	SET and MYND domain-containing protein 1	DNA binding; transcription factor activity; transcription cofactor activity	nucleobase, nucleoside, nucleotide and nucleic acid metabolic process

46	2.48	**KTN1**	Kinectin	structural constituent of cytoskeleton	intracellular protein transport; **cellular component morphogenesis**

47	2.48	PJA2	E3 ubiquitin-protein ligase Praja2	DNA binding; transcription factor activity	

48	2.48	SRPK1	Serine/threonine-protein kinase SRPK1	kinase activity	immune system process; mitosis; cell surface receptor linked signal transduction; intracellular signaling cascade; carbohydrate metabolic process; protein metabolic process; cell motion; mitosis; signal transduction; segment specification; ectoderm development; mesoderm development; embryonic development; nervous system development; response to stress

49	2.47	ANKS3	Ankyrin repeat and SAM domain-containing protein 3		

50	2.47	C20orf26	Uncharacterized protein C20orf26		

51	2.47	TREML2	Trem-like transcript 2 protein		

52	2.46	TAB2	Mitogen-activated protein kinase kinase kinase 7-interacting protein 2	DNA binding; transcription factor activity	nucleobase, nucleoside, nucleotide and nucleic acid metabolic process

53	2.45	C19orf6	Membralin	structural molecule activity	

54	2.45	EPB41L2	Band 4.1-like protein 2		

55	2.44	CCDC71	Coiled-coil domain-containing protein 71		

56	2.44	FGD1	FYVE, RhoGEF and PH domain-containing protein 1	protein binding; small GTPase regulator activity; guanyl-nucleotide exchange factor activity	mesoderm development; skeletal system development

57	2.44	**VCAM1**	Vascular cell adhesion protein 1	hydrolase activity, acting on ester bonds; phosphatase activity; receptor activity	immune system process; muscle contraction; induction of apoptosis; cell cycle; cell surface receptor linked signal transduction; cell-cell signaling; **cell-cell adhesion**; protein metabolic process; cell motion; cell cycle; signal transduction; ectoderm development; mesoderm development; angiogenesis; nervous system development; muscle organ development

58	2.42	ANKRA2	Ankyrin repeat family A protein 2	DNA binding; transcription factor activity	antigen processing and presentation of peptide or polysaccharide antigen via MHC class II; cellular defense response

59	2.42	KCNK17	Potassium channel subfamily K member 17	cation transmembrane transporter activity; voltage-gated potassium channel activity; cation channel activity	neurological system process; cation transport

60	2.42	TXNDC15	Thioredoxin domain-containing protein 15		

61	2.41	TBPL2	TATA box-binding protein-like protein 2	DNA binding; transcription factor activity	nucleobase, nucleoside, nucleotide and nucleic acid metabolic process

62	2.40	ALDH3A2	Fatty aldehyde dehydrogenase	oxidoreductase activity	carbohydrate metabolic process; nucleobase, nucleoside, nucleotide and nucleic acid metabolic process; cellular amino acid and derivative metabolic process

63	2.40	LAMP3	Lysosome-associated membrane glycoprotein 3		lysosomal transport; intracellular protein transport; protein metabolic process

64	2.40	PNLIP	Pancreatic triacylglycerol lipase	hydrolase activity, acting on ester bonds	lipid metabolic process

65	2.37	C17orf28	UPF0663 transmembrane protein C17orf28		

66	2.36	GPR137	Integral membrane protein GPR137		

67	2.35	WARS	Tryptophanyl-tRNA synthetase, cytoplasmic	aminoacyl-tRNA ligase activity	protein metabolic process

68	2.34	C11orf35	Uncharacterized protein C11orf35		

69	2.34	SCML2	Sex comb on midleg-like protein 2	DNA binding; chromatin binding; transcription factor activity	cell cycle; nucleobase, nucleoside, nucleotide and nucleic acid metabolic process; cell cycle; organelle organization; establishment or maintenance of chromatin architecture; ectoderm development; mesoderm development; nervous system development

70	2.33	GNL1	Guanine nucleotide-binding protein-like 1	GTPase activity; nucleic acid binding; receptor binding	intracellular protein transport; cell surface receptor linked signal transduction; intracellular signaling cascade; nucleobase, nucleoside, nucleotide and nucleic acid metabolic process; signal transduction

71	2.33	MORN1	MORN repeat-containing protein 1	kinase activity	cell surface receptor linked signal transduction; signal transduction

72	2.33	TMEM149	Transmembrane protein 149		

73	2.32	AK4	Adenylate kinase isoenzyme 4, mitochondrial	kinase activity	nucleobase, nucleoside, nucleotide and nucleic acid metabolic process

74	2.32	TOMM34	Mitochondrial import receptor subunit TOM34		immune system process; protein metabolic process; response to stress

75	2.32	ZNHIT6	Zinc finger HIT domain-containing protein 6		

76	2.31	BTG1	Protein BTG1		cell cycle; intracellular signaling cascade; signal transduction

77	2.30	GRAMD1A	GRAM domain-containing protein 1A		

78	2.30	GRIK5	Glutamate receptor, ionotropic kainate 5	glutamate receptor activity; ligand-gated ion channel activity	neurological system process; cation transport; cell surface receptor linked signal transduction; synaptic transmission; signal transduction; cell-cell signaling

79	2.30	TDRKH	Tudor and KH domain-containing protein	hydrolase activity, acting on ester bonds;nucleic acid binding	nucleobase, nucleoside, nucleotide and nucleic acid metabolic process

80	2.30	TGIF1	Homeobox protein TGIF1	DNA-directed RNA polymerase activity; DNA binding; transcription factor activity	nucleobase, nucleoside, nucleotide and nucleic acid metabolic process; ectoderm development; nervous system development

81	2.29	FAM81B	Protein FAM81B		

82	2.29	MRPL9	39S ribosomal protein L9, mitochondrial	structural constituent of ribosome; nucleic acid binding	protein metabolic process

83	2.28	ANKRD46	Ankyrin repeat domain-containing protein 46		

84	2.27	INSM1	Insulinoma-associated protein 1		nucleobase, nucleoside, nucleotide and nucleic acid metabolic process

85	2.27	PBX1	Pre-B-cell leukemia transcription factor 1	DNA-directed RNA polymerase activity; DNA binding; transcription factor activity	nucleobase, nucleoside, nucleotide and nucleic acid metabolic process; ectoderm development; mesoderm development; nervous system development; hemopoiesis

86	2.27	TPRG1	Tumor protein p63-regulated gene 1 protein		

87	2.26	SEPT4	Septin-4	GTPase activity;structural constituent of cytoskeleton; protein binding	mitosis; cytokinesis

88	2.25	FAM159B	UPF0514 membrane protein FAM159B		

89	2.24	AKAP8	A-kinase anchor protein 8		mitosis; chromosome segregation

90	2.24	FAM83A	Protein FAM83A		

91	2.24	**MAG**	Myelin-associated glycoprotein	receptor activity; structural constituent of myelin sheath; receptor binding	B cell mediated immunity; cell surface receptor linked signal transduction; **cell-cell adhesion**; signal transduction; ectoderm development; nervous system development; response to stimulus

92	2.24	SCN9A	Sodium channel protein type 9 subunit alpha	cation transmembrane transporter activity; voltage-gated sodium channel activity; cation channel activity	neuronal action potential propagation; cation transport

93	2.23	ASTN1	Astrotactin-1		

94	2.23	RAB7L1	Ras-related protein Rab-7 L1	GTPase activity; protein binding	neurotransmitter secretion; intracellular protein transport; exocytosis; endocytosis; cell cycle; cell surface receptor linked signal transduction; intracellular signaling cascade; signal transduction

95	2.22	RASA3	Ras GTPase-activating protein 3	protein binding; small GTPase regulator activity	signal transduction

96	2.22	TMEM59L	Transmembrane protein 59-like		

97	2.21	CACNB3	Voltage-dependent L-type calcium channel subunit beta-3	cation transmembrane transporter activity; voltage-gated calcium channel activity; cation channel activity	muscle contraction; neurotransmitter secretion; synaptic transmission; cell-cell signaling

98	2.21	PLCD3	1-phosphatidylinositol-4,5-bisphosphate phosphodiesterase delta-3	hydrolase activity, acting on ester bonds; calcium ion binding	cell surface receptor linked signal transduction; lipid metabolic process; signal transduction

99	2.21	PLCH1	1-phosphatidylinositol-4,5-bisphosphate phosphodiesterase eta-1	hydrolase activity, acting on ester bonds; calcium ion binding; receptor binding; small GTPase regulator activity; guanyl-nucleotide exchange factor activity	cell surface receptor linked signal transduction; intracellular signaling cascade; lipid metabolic process; signal transduction

100	2.21	TULP1	Tubby-related protein 1		visual perception; sensory perception; ectoderm development; nervous system development

101	2.20	EFCAB6	EF-hand calcium-binding domain-containing protein 6;EFCAB6	calcium ion binding; receptor binding; calmodulin binding; enzyme regulator activity	cation transport; cell cycle;signal transduction; cell cycle; signal transduction

102	2.19	**PTPN6**	Tyrosine-protein phosphatase non-receptor type 6	hydrolase activity, acting on ester bonds; phosphatase activity; receptor activity	immune system process; intracellular protein transport; mitosis; cell surface receptor linked signal transduction; intracellular signaling cascade; **cell-matrix adhesion**; **cell-cell adhesion; **protein metabolic process; cytokinesis; cell motion; signal transduction; nervous system development; cellular glucose homeostasis

103	2.18	GPR155	Integral membrane protein GPR155	receptor activity	

104	2.18	PSPN	Persephin	receptor binding	neurological system process; cell surface receptor linked signal transduction; cell-cell signaling; signal transduction; ectoderm development; nervous system development

105	2.18	STAM2	Signal transducing adapter molecule 2	transmembrane transporter activity; protein binding; kinase activator activity; kinase regulator activity	lysosomal transport; intracellular protein transport; endocytosis; intracellular signaling cascade; signal transduction

106	2.17	AGXT2L2	Alanine--glyoxylate aminotransferase 2-like 2	transaminase activity	visual perception; sensory perception; vitamin biosynthetic process; cellular amino acid and derivative metabolic process

The list of up-regulated genes in cancer cells grown as a co-culture with carcinoma-associated fibroblasts (CAFs). Gene ID, name, molecular function and biological process are listed according to the Panther Database classification www.pantherdb.org. The analyzed genes were significantly up-regulated at the level of *p *< 0.05, fold change > 2.0.

**Table 3:** Down-regulated genes in cancer cells grown as a co-culture with CAFs

No	Fold change	Gene ID	Gene name	Molecular function	Biological process
1	2.17	EPS8L1	Epidermal growth factor receptor kinase substrate 8-like protein 1		intracellular signaling cascade; cell motion; signal transduction

2	2.17	OR4X1	Olfactory receptor 4X1		

3	2.18	C2orf61	Uncharacterized protein C2orf61		

4	2.18	DSN1	Kinetochore-associated protein DSN1 homolog		

5	2.18	PFAS	Phosphoribosylformylglycinamidine synthase	ligase activity	nucleobase, nucleoside, nucleotide and nucleic acid metabolic process

6	2.18	SLC5A2	Sodium/glucose cotransporter 2	carbohydrate transmembrane transporter activity; cation transmembrane transporter activity	cation transport; extracellular transport; amino acid transport; carbohydrate metabolic process; cellular amino acid and derivative metabolic process

7	2.18	TMEM138	Transmembrane protein 138		

8	2.19	FOLR1	Folate receptor alpha	receptor activity;transmembrane transporter activity	vitamin transport

9	2.19	MFN2	Mitofusin-2	hydrolase activity, acting on ester bonds; phosphatase activity	intracellular protein transport; organelle organization; mitochondrion organization

10	2.19	NDRG3	Protein NDRG3		

11	2.19	UNC13D	Protein unc-13 homolog D		intracellular protein transport; exocytosis

12	2.20	GOLPH3	Golgi phosphoprotein 3		

13	2.20	SLC22A13	Solute carrier family 22 member 13	ATPase activity, coupled to transmembrane movement of substances; ligase activity; carbohydrate transmembrane transporter activity; cation transmembrane transporter activity	cation transport; anion transport; extracellular transport; carbohydrate transport; carbohydrate metabolic process

14	2.20	USP54	Inactive ubiquitin carboxyl-terminal hydrolase 54	ubiquitin-protein ligase activity	protein metabolic process

15	2.22	NMI	N-myc-interactor	DNA binding; transcription factor activity; transcription cofactor activity	response to interferon-gamma; intracellular signaling cascade; signal transduction; cellular defense response

16	2.22	PDCD1	Programmed cell death protein 1		

17	2.22	SNRPN	Small nuclear ribonucleoprotein-associated protein N	RNA splicing factor activity, transesterification mechanism; RNA binding	nucleobase, nucleoside, nucleotide and nucleic acid metabolic process

18	2.23	SPSB1	SPRY domain-containing SOCS box protein 1		

19	2.24	ADAMTS15	A disintegrin and metalloproteinase with thrombospondin motifs 15	peptidase activity; protein binding; peptidase inhibitor activity	fertilization; signal transduction; cell-matrix adhesion; cell-cell adhesion; protein metabolic process; signal transduction; ectoderm development; mesoderm development; skeletal system development; angiogenesis; nervous system development; muscle organ development

20	2.24	MTRF1L	Peptide chain release factor 1-like, mitochondrial	translation factor activity, nucleic acid binding; translation release factor activity	protein metabolic process

21	2.24	TOMM7	Mitochondrial import receptor subunit TOM7 homolog	transmembrane transporter activity	intracellular protein transport

22	2.25	ATG7	Autophagy-related protein 7	ligase activity	intracellular signaling cascade; coenzyme metabolic process; protein metabolic process; signal transduction

23	2.25	C19orf52	Uncharacterized protein C19orf52		

24	2.25	USF2	Upstream stimulatory factor 2	DNA binding; transcription factor activity	nucleobase, nucleoside, nucleotide and nucleic acid metabolic process; lipid metabolic process

25	2.26	POGK	Pogo transposable element with KRAB domain	DNA binding	nucleobase, nucleoside, nucleotide and nucleic acid metabolic process; ectoderm development; nervous system development

26	2.26	ZNF804B	Zinc finger protein 804B		

27	2.27	CD274	Programmed cell death 1 ligand 1	ubiquitin-protein ligase activity; receptor activity; DNA binding; receptor binding; transcription factor activity; transcription cofactor activity	immune system process; neurotransmitter secretion; intracellular protein transport; exocytosis; signal transduction; synaptic transmission; nucleobase, nucleoside, nucleotide and nucleic acid metabolic process; protein metabolic process; cell-cell signaling; organelle organization; establishment or maintenance of chromatin architecture; mesoderm development; mammary gland development; response to stress; cellular defense response

28	2.27	FAM132B	Protein FAM132B		

29	2.27	STK32C	Serine/threonine-protein kinase 32 C	kinase activity	cell cycle; intracellular signaling cascade; protein metabolic process; cell cycle; signal transduction

30	2.28	CADM4	Cell adhesion molecule 4	receptor activity	

31	2.28	GLIPR2	Golgi-associated plant pathogenesis-related protein 1		immune system process

32	2.28	GPR160	Probable G-protein coupled receptor 160		

33	2.29	GALNT2	Polypeptide N-acetylgalactosaminyltransferase 2 soluble form	transferase activity, transferring glycosyl groups	carbohydrate metabolic process; protein metabolic process

34	2.29	KRT20	Keratin, type I cytoskeletal 20	structural constituent of cytoskeleton	cellular component morphogenesis; cellular component morphogenesis; ectoderm development; cellular component morphogenesis

35	2.29	PTPN6	Tyrosine-protein phosphatase non-receptor type 6	hydrolase activity, acting on ester bonds; phosphatase activity; receptor activity	immune system process; intracellular protein transport; mitosis; cell surface receptor linked signal transduction; intracellular signaling cascade; cell-matrix adhesion; cell-cell adhesion; protein metabolic process; cytokinesis; cell motion; mitosis; signal transduction; nervous system development; cellular glucose homeostasis

36	2.29	SOHLH1	Spermatogenesis- and oogenesis-specific basic helix-loop-helix-containing protein 1		

37	2.31	CENPM	Centromere protein M		

38	2.31	HPCAL1	Hippocalcin-like protein 1	calcium ion binding; calmodulin binding; small GTPase regulator activity	visual perception; sensory perception; cell surface receptor linked signal transduction; signal transduction

39	2.31	TMEM81	Transmembrane protein 81		

40	2.32	HOXA1	Homeobox protein Hox-A1	DNA binding; transcription factor activity	female gamete generation; nucleobase, nucleoside, nucleotide and nucleic acid metabolic process; segment specification; ectoderm development; gut mesoderm development; embryonic development; skeletal system development; angiogenesis; nervous system development; muscle organ development

41	2.32	SMO	Smoothened homolog	G-protein coupled receptor activity; receptor binding	cell surface receptor linked signal transduction; cell-cell signaling

42	2.33	LAMP2	Lysosome-associated membrane glycoprotein 2		lysosomal transport; intracellular protein transport; protein metabolic process

43	2.33	RNF121	RING finger protein 121	ubiquitin-protein ligase activity	protein metabolic process

44	2.34	PLA2G2E	Group IIE secretory phospholipase A2	hydrolase activity, acting on ester bonds	signal transduction; lipid metabolic process; signal transduction

45	2.34	POU6F2	POU domain, class 6, transcription factor 2	DNA binding; transcription factor activity	nucleobase, nucleoside, nucleotide and nucleic acid metabolic process

46	2.34	PPP2R4	Serine/threonine-protein phosphatase 2A regulatory subunit B'	protein binding;phosphatase activator activity; phosphatase regulator activity	protein metabolic process

47	2.34	STX5	Syntaxin-5	SNAP receptor activity	neurotransmitter secretion; intracellular protein transport; exocytosis; endocytosis; synaptic transmission; cell-cell signaling

48	2.35	DMC1	Meiotic recombination protein DMC1/LIM15 homolog	hydrolase activity; DNA binding	immune system process; meiosis; nucleobase, nucleoside, nucleotide and nucleic acid metabolic process; meiosis; response to stress

49	2.35	ERRFI1	ERBB receptor feedback inhibitor 1		signal transduction;signal transduction

50	2.36	TSPYL4	Testis-specific Y-encoded-like protein 4	protein binding; phosphatase inhibitor activity; phosphatase regulator activity	apoptosis; cell cycle; nucleobase, nucleoside, nucleotide and nucleic acid metabolic process; protein metabolic process; cell cycle; organelle organization; establishment or maintenance of chromatin architecture

51	2.37	USF1	Upstream stimulatory factor 1	DNA binding; transcription factor activity	nucleobase, nucleoside, nucleotide and nucleic acid metabolic process; lipid metabolic process

52	2.40	ARFGAP3	ADP-ribosylation factor GTPase-activating protein 3	nucleic acid binding; protein binding; small GTPase regulator activity	cell surface receptor linked signal transduction; cell adhesion

53	2.40	CNBP	Cellular nucleic acid-binding protein	nucleic acid binding	nucleobase, nucleoside, nucleotide and nucleic acid metabolic process; lipid metabolic process

54	2.40	NKD1	Protein naked cuticle homolog 1		

55	2.41	MRPL51	39S ribosomal protein L51, mitochondrial;	structural constituent of ribosome; nucleic acid binding	protein metabolic process

56	2.41	OPRK1	Kappa-type opioid receptor	G-protein coupled receptor activity	sensory perception; cell surface receptor linked signal transduction; synaptic transmission; cell motion; signal transduction; cell-cell signaling

57	2.41	PTX3	Pentraxin-related protein PTX3		immune response; response to stress; defense response to bacterium

58	2.42	GPRC5B	G-protein coupled receptor family C group 5 member B	G-protein coupled receptor activity	cell surface receptor linked signal transduction; signal transduction

59	2.42	NGLY1	Peptide-N(4)-(N-acetyl-beta-glucosaminyl)asparagine amidase	hydrolase activity	protein metabolic process

60	2.43	CAPN12	Calpain-12	peptidase activity; calcium ion binding; calmodulin binding; calcium-dependent phospholipid binding	induction of apoptosis; intracellular signaling cascade; protein metabolic process; signal transduction

61	2.43	POLD1	DNA polymerase delta catalytic subunit	DNA-directed DNA polymerase activity; hydrolase activity, acting on ester bonds; nucleic acid binding	cell cycle; nucleobase, nucleoside, nucleotide and nucleic acid metabolic process; cell cycle

62	2.44	TBC1D8	TBC1 domain family member 8	hydrolase activity; protein binding; small GTPase regulator activity	intracellular protein transport; exocytosis; cellular component morphogenesis

63	2.45	DHRS11	Dehydrogenase/reductase SDR family member 11	oxidoreductase activity	visual perception; sensory perception; lipid metabolic process

64	2.46	LZTR1	Leucine-zipper-like transcriptional regulator 1	structural constituent of cytoskeleton; DNA binding; chromatin binding; protein binding; small GTPase regulator activity; transcription factor activity	spermatogenesis; immune system process; intracellular protein transport; vesicle-mediated transport; cell cycle; nitrogen compound metabolic process; nucleobase, nucleoside, nucleotide and nucleic acid metabolic process; protein metabolic process

65	2.46	OR6V1	Olfactory receptor 6 V1		

66	2.47	C12orf65	Uncharacterized protein C12orf65	translation factor activity, nucleic acid binding; translation release factor activity	protein metabolic process

67	2.47	C2orf56	Protein midA homolog, mitochondrial		

68	2.47	RBM42	RNA-binding protein 42	RNA splicing factor activity, transesterification mechanism; DNA binding; RNA binding	spermatogenesis; neurological system process; cell cycle; nucleobase, nucleoside, nucleotide and nucleic acid metabolic process; protein metabolic process; ectoderm development; nervous system development

69	2.48	DPM2	Dolichol phosphate-mannose biosynthesis regulatory protein		

70	2.48	TDRD12	Tudor domain-containing protein 12	RNA helicase activity; translation factor activity, nucleic acid binding; translation initiation factor activity	nucleobase, nucleoside, nucleotide and nucleic acid metabolic process; protein metabolic process

71	2.53	CCDC85B	Coiled-coil domain-containing protein 85B		

72	2.54	MATN1	Cartilage matrix protein	extracellular matrix structural constituent	immune system process; sensory perception of sound;sensory perception; signal transduction; cell-cell adhesion; cellular component morphogenesis; mesoderm development; skeletal system development; blood coagulation

73	2.55	FLYWCH1	FLYWCH-type zinc finger-containing protein 1		

74	2.55	PLA2G2D	Group IID secretory phospholipase A2	hydrolase activity, acting on ester bonds	signal transduction; lipid metabolic process; signal transduction

75	2.56	TMEM38A	Trimeric intracellular cation channel type A		

76	2.58	RGS11	Regulator of G-protein signaling 11	protein binding;small GTPase regulator activity	cell surface receptor linked signal transduction; signal transduction; dorsal/ventral axis specification

77	2.61	BAHD1	Bromo adjacent homology domain-containing 1 protein	DNA binding	nucleobase, nucleoside, nucleotide and nucleic acid metabolic process

78	2.67	CHD5	Chromodomain-helicase-DNA-binding protein 5	DNA helicase activity; nucleic acid binding	nucleobase, nucleoside, nucleotide and nucleic acid metabolic process; organelle organization; establishment or maintenance of chromatin architecture

79	2.67	CHD5	Tryptophan-rich protein;	structural constituent of ribosome; nucleic acid binding	protein metabolic process

80	2.67	CYP39A1	Cytochrome P450 39A1	oxidoreductase activity	respiratory electron transport chain; lipid metabolic process

81	2.68	SUDS3	Sin3 histone deacetylase corepressor complex component SDS3		cell cycle

82	2.69	C16orf62	UPF0505 protein C16orf62		

83	2.69	FLOT2	Flotillin-2		intracellular protein transport; vesicle-mediated transport

84	2.69	NINJ1	Ninjurin-1		neurological system process; cell adhesion; cell adhesion; ectoderm development; nervous system development

85	2.70	FAM84A	Protein FAM84A		

86	2.71	PAF1	RNA polymerase II-associated factor 1 homolog		

87	2.71	PAF1	Peroxisome assembly factor 1		protein metabolic process

88	2.77	POLR2F	DNA-directed RNA polymerases I, II, and III subunit	DNA-directed RNA polymerase activity; nucleic acid binding	nucleobase, nucleoside, nucleotide and nucleic acid metabolic process

89	2.78	C12orf34	Uncharacterized protein C12orf34		

90	2.80	GGT6	Gamma-glutamyltransferase 6 light chain	acyltransferase activity; peptidase activity	cellular amino acid and derivative metabolic process; protein metabolic process

91	2.83	TSC22D4	TSC22 domain family protein 4	DNA binding; transcription factor activity	nucleobase, nucleoside, nucleotide and nucleic acid metabolic process

92	2.84	CLRN2	Clarin-2		

93	2.92	KCNS1	Potassium voltage-gated channel subfamily S member 1	cation transmembrane transporter activity; voltage-gated potassium channel activity; cation channel activity	muscle contraction; blood circulation; neuronal action potential propagation; cation transport; signal transduction; synaptic transmission; signal transduction; cell-cell signaling

94	2.94	OR51T1	Olfactory receptor 51 T1		

95	2.96	OR51I1	Olfactory receptor 51I1		

96	3.01	ZNF135	Zinc finger protein 135	DNA binding;transcription factor activity	nucleobase, nucleoside, nucleotide and nucleic acid metabolic process

97	3.03	QDPR	Dihydropteridine reductase	oxidoreductase activity	cellular amino acid and derivative metabolic process

98	3.11	C9	Complement component C9b	peptidase activity; receptor activity	complement activation; signal transduction; cell-cell adhesion; protein metabolic process; signal transduction; response to stimulus

99	3.16	VDAC1	Voltage-dependent anion-selective channel protein 1	voltage-gated ion channel activity; anion channel activity	anion transport

100	3.85	FDX1L	Adrenodoxin-like protein, mitochondrial	oxidoreductase activity	respiratory electron transport chain; ferredoxin metabolic process; vitamin metabolic process; lipid metabolic process; protein metabolic process

The list of down-regulated genes in cancer cells grown as a co-culture with carcinoma-associated fibroblasts (CAFs). Gene ID, name, molecular function and biological process are listed according to the Panther Database classification www.pantherdb.org. The analyzed genes were significantly down-regulated at the level of *p *< 0.05, fold change > 2.0.

The PANTHER binomial statistics tool to compare classifications of multiple clusters of lists to a reference list of *Canis familiaris *genes allowed us to statistically determine over-manifestation of PANTHER biological process and pathways classification categories. The PANTHER biological process analysis revealed that most of the up-regulated genes in cancer cells grown as a co-culture with the CAFs were involved in: cell surface receptor linked signal transduction (*p *= 9.05E-03), lysosomal transport (*p *= 9.2E-03), developmental process (*p *= 3.64E-02), antigen processing and presentation (*p *= 4.22E-02), signal transduction (*p *= 4.35E-02), cell communication (*p *= 4.55E-02), nervous system development (*p *= 4.97E-02).

Because our concern involved interactions between cancer cells and carcinoma associated fibroblasts which could predispose cancer to metastasis, we specifically focused on the up-regulated genes involved in cell adhesion and cellular morphogenesis: QRICH2, CHAD, FOXQ1, PCSK6, CLEC7A, SH3BP1, NEFM, PCDH19, LPHN2, DSP, KTN1, VCAM1, MAG, and PTPN6 (Table 2). Interestingly, the gene expression analysis also revealed an up-regulation of 24 genes involved in developmental processes such as: a mammary gland development, a mesoderm development, an ectoderm development, a skeletal system development, a nervous system development, an embryonic development, a heart development, and a muscle-organ development. The pathway analysis revealed that significantly over-manifested were: the salvage pyrimidine ribonucleotides (*p *= 02.87E-03), the oxytocin receptor mediated signaling pathway (*p *= 4.76E-03), the thyrotropin-releasing hormone receptor signaling (*p *= 5.21E-03), the 5HT2 type receptor mediated signaling pathway (*p *= 6.99E-03), the metabotropic glutamate receptor III (*p *= 8.14E-03), the metabotropic glutamate receptor group II (*p *= 3.33E-02), the metabotropic glutamate receptor I (*p *= 1.75E-02), the Beta 2 and Beta1 adrenergic receptor signaling (*p *= 2.54E-02) and the histamine H1 receptor mediated signaling (*p *= 2.87E-02).

Among the down-regulated genes no significant pathways or biological processes and their over-manifestation were found (Table 3) comparing to a reference list of *Canis familiaris *genes.

### The results were confirmed at mRNA level using real-time qPCR analysis

**Figure 5:**
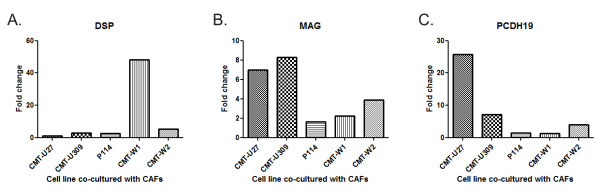
**Selected genes expression assessed using real-time qPCR**. Expression of randomly selected genes in canine mammary cancer lines growing as a monoculture and co-culture with CAFs. The changes in gene expression differed highly significant (*p *< 0.001, *N = *3).

For the purposes of the microarray data validation, we have randomly selected 3 of all the genes that may play the most important role in the cancer cells-CAFs interactions: PCDH19, DSP and MAG. Real-time qPCR results showed similar trends in gene expression changes as were observed in microarray studies (Figure 5).

### MUC1 expression detection in cancer cell lines

**Figure 6:**
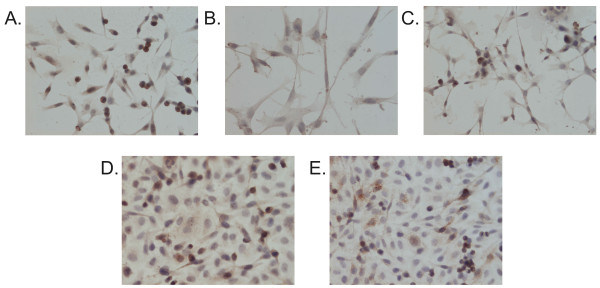
**MUC1 expression in canine mammary cancer cell lines**. MUC1 expression (brown color) in canine mammary cancer cell lines: A. CMT-U27, B. CMT-U309, C. P114, D. CMT-W1 and E. CMT-W2. The pictures were obtained using Olympus BX60 microscope (at the magnification of 200×).

Because the MAG gene up-regulation was found in cancer cells grown as a co-culture with the CAFs, the expression of MUC1 (which binds MAG) was examined immunohistochemically (Figure 6). The MUC1 expression was confirmed in all of the examined cell lines.

## Discussion

Canine mammary cancers in bulk arise from epithelial cells. Several genetic alterations have been detected, that may predispose these cells to the malignant transformation [10-12]. However, researches over the last few years suggested that concomitant changes also occur in stromal cells that form the tumour microenvironment [1,2]. The hypothesis of stromal cells involvement in tumorigenesis is based on a study of embryological development where interactions between various cells are necessary for programming and maintaining epithelial structure and function. The embryonic epithelial and stromal cells of mesenchymal origin engage in a molecular dialogue that ensures the proper organ development and function [3].

The study showed in cancer cells after a co-culture with CAFs an up-regulation of 23 genes (Table 2) involved in developmental processes (a nervous system development, an embryonic development, a mesoderm and ectoderm development).

The involvement of fibroblasts in the malignant transformation of epithelial cells has previously been documented [3,26-28]. Moreover, the histology and growth characteristics of CAFs were found different from those of the fibroblasts associated with normal epithelial cells [3]. Mishra et al. [29] have proposed bidirectional cross-talk between the CAFs and the cancer cells which release proteins that increase the fibroblasts ability to secrete a variety of tumour-promoting factors, which then act back on the malignant cells to change their gene expression and promote their proliferative, migratory, and invasive properties. On the other hand, other studies showed that only direct contact of fibroblasts with cancer cells is able to cause changes in their gene expression and biology [30-32].

So far several papers have been published about gene expression in tumour microenvironment. Most of them describe gene expression in fibroblasts, but not in cancer cells, however there are some papers available about the changes in gene expression in cancer cells [33-36]. These reports indicated up-regulation of genes involved in angiogenesis, EMT and migration in cancer cells grown with fibroblasts. Surprisingly, some of the genes identified, even though functionally identical turned out to be of different names. The studies have been conducted using various cancer models (various species) and various cell lines, so the differences are possible.

The results of the study hereby revealed increased expression of 13 genes involved in cell adhesion (Table 2) among cancer cells co-cultured with the CAFs. As much as 10 of them are involved in developmental processes as well. These genes seem to be particularly significant because the cell adhesion is responsible for tumour progression and metastasis, detachment from the primary tumour and spreading to the circulatory system. Moreover, the up-regulated genes responsible for adhesion are by rule involved in angiogenesis and lymphangiogenesis. For example, the vascular cell adhesion molecule-1 (VCAM-1) up-regulated in cancer cells grown as a co-culture with CAFs may be involved in tumor progression and metastasis particularly via lymphangiogenesis promotion [37,38]. It also has previously been demonstrated that the VCAM-1 plays a crucial role in the endothelial-carcinoma cell adhesion [38].

The study also revealed an up-regulation of desmoplakin (DSP), which is a key component of cellular adhesion junctions known as desmosomes. These junctions are found at contact sites between endothelial cells that form capillaries, thus DSP play a role in *de novo *capillary formation and branching during tumourigenesis, embryonic development and cardiovascular development [39]. Moreover, desmoplakin isoform 2 was only detected in tumours associated with a poor clinical outcome. It may suggest its potentially specific function in the regulation of cancer cells proliferation, differentiation, invasion and metastasis [40,41].

Moreover, desmosomes may also be important in the epithelium-mesenchymal transition (EMT). The epithelium-mesenchymal transition is an indispensable mechanism for morphogenesis during embryonic development, and is implicated in conversion of early-stage tumours into invasive cancers. During EMT, epithelial cells undergo changes in morphology and acquire the migratory and invasive characteristics of mesenchymal cells [30]. EMT is also promoted by the FOXQ1, another up-regulated gene in cancer cells grown under co-culture conditions with the CAFs [42]. It also increases expression of several junction proteins promoting cancer cells to gain the stem-cell-like properties and ensuring resistance to apoptosis [42-44]. Moreover, the down regulation of keratin 20 (Table 3) in cancer cells following the co-culture with the CAFs may indicate the EMT induction [45,46].

Interestingly, another up-regulated gene in cancer cells grown with the CAFs, which contributes to cancer invasion is myelin-associated glycoprotein (MAG) that binds to the oncogenic glycoprotein MUC1 [47]. Swanson et al. [47] described an interaction between the MUC1 and the MAG in cancers that invade perineurally, including prostate, salivary, and breast carcinomas. Furthermore, breast cancers may metastasize to the brain where the MAG is abundantly expressed. Interactions between the MUC1 and the MAG have not fully been defined yet. We confirmed the MUC1 expression in all of the examined cell lines (Figure 6). Thus, based on our own observations and those of Swanson et al. [47], we suppose that the MAG up-regulation in cancer cells grown with the CAFs and its binding to the MUC1 may contribute to the adhesion between tumour cells and Schwann cells promoting metastasis to the nervous system.

We also found a down-regulation of 5 key genes associated with adhesion. Subject literature suggests 3 of them play a role in cancer development. The down-regulation of these genes is associated with poor prognosis and cancer metastases. One of these genes is the ADAMTS15 (a disintegrin and metalloproteinase with thrombospondin motif 15) which is an anti-angiogenic factor [48]. Our study also revealed a down-regulation of the CADM4. Nagata et al. [49] found decrease in the CADM4 expression in most of renal cell carcinomas and the cancer cell lines. Moreover, the CADM4 expression was decreased in carcinomas with vascular infiltration, suggesting that loss of the CADM4 is involved in tumour angiogenesis and invasion. We have also found a down-regulation of the MATN1 gene which has been defined an angiogenesis inhibitor [50].

In the current study we showed a significant over-manifestation of genes involved in the oxytocin receptor mediated signaling pathway, the thyrotropin-releasing hormone receptor signaling, the Beta 2 and Beta1 adrenergic receptor signaling, and the histamine H1 receptor mediated signaling in cancer cells grown with the CAFs. Entschladen et al. [51] described the role of neurotransmitters in cancer progression and metastasis. They found that similarly to chemokines, neurotransmitters are regulators of cell migration. Sadly though, we noticed that only a few results are available on the expression of neurotransmitter receptors in tumour tissues. Among them the best understood is the role of catecholamines in carcinogenesis and tumour progression. These are the stress hormones, whereas stress in turn is a major risk factor for the development of cancer. Norepinephrine has been shown to strongly induce the migration of tumor cells [52,53], whereas epinephrine was found a modulator for the carcinogenesis in the lung [54].

An interesting gene in cancer cells grown as a co-culture with the CAFs is the up-regulated protocadherin 19 (PCDH19) (Table 2). Up-to-date there is no information available on the involvement of this gene in tumor progression or metastasis. However, a PCDH19 mutation was found to be responsible for epilepsy and mental retardation confined to females (EFMR) [55]. There has been an on-going debate about the relationship between epilepsy and cancer. It has been hypothesized that the incidence of cancer is increased in people with epilepsy owing to the cancer promotion by antiepileptic drugs [56]. Perhaps the increased risk of cancer in epileptic patients is caused by the PCDH19 mutation and over-expression, not however related to drugs toxicity. This hypothesis requires further studies.

## Conclusions

The results of the current study showed that the co-culture of cancer cells and the CAFs caused significant changes in expression of genes involved in adhesion, angiogenesis and the EMT that take part in developmental processes.

## Competing interests

The authors declare that they have no competing interests.

## Authors' contributions

MK: research design, experimental design, FACS cell sorting, CAFs isolation, manuscript and figures preparation; KP: cell culturing, CAFs isolation, RNA isolation, microarray analyses; KS: real-time qPCR; HM statistical analysis of microarray experiment; ID: histopathological examination of tumour sample and CAFs; EM: immunohistochemical examination; MJ: microarray analysis; TM: manuscript preparation. All authors read and approved the final manuscript.
